# Subfoveal choroidal thickness in a general elderly population; Tehran geriatric eye study

**DOI:** 10.1186/s12886-024-03401-0

**Published:** 2024-03-27

**Authors:** Alireza Hashemi, Payam Nabovati, Abolghasem Mortazavi, Hassan Hashemi, Mehdi Khabazkhoob

**Affiliations:** 1 Noor Ophthalmology Research Center, Noor Eye Hospital, Tehran, Iran; 2 Rehabilitation Research Center, Department of Optometry, School of Rehabilitation Sciences, Iran University of Medical Sciences, Tehran, Iran; 3 Department of Neurosurgery, Sina Hospital, Tehran University of Medical Sciences, Tehran, Iran; 4 Noor Research Center for Ophthalmic Epidemiology, Noor Eye Hospital, Tehran, Iran; 5 Department of Basic Sciences, School of Nursing and Midwifery, Shahid Beheshti University of Medical Sciences, Tehran, Iran

**Keywords:** Subfoveal choroidal thickness, Cross-sectional study, Elderly, SD-OCT

## Abstract

**Purpose:**

To determine the distribution of subfoveal choroidal thickness (SFCT) and its associated demographic, ocular, and systemic factors in an elderly population.

**Methods:**

This report is part of the Tehran Geriatric Eye Study (TGES); a population-based cross-sectional study that was conducted on the urban elderly population of Tehran, aged 60 years and above using multi-stage stratified random cluster sampling. Choroidal imaging was performed using Spectralis SD-OCT with enhanced depth imaging mode.

**Results:**

The average SFCT was 265.3 ± 25.9 μm (95% CI: 262.8-267.7) in the whole sample. According to the multiple generalized estimating equation (GEE) model, pseudophakia had a statistically significant direct relationship with SFCT (coefficient = 5.69), and history of cerebrovascular accident (CVA) was significantly inversely related to SFCT (coefficient=-4.77). Moreover, there was a significant interaction between age and sex in the average SFCT so that with increasing age, the SFCT increased in men and decreased in women.

**Conclusion:**

The normal values of SFCT in the present study can be used as a reference database for clinical and research purposes. Age-sex interaction, pseudophakia, and history of CVA were significantly associated with SFCT in the elderly population. It is recommended that these factors be taken into account when interpreting SFCT data.

## Introduction

The choroid plays a vital role in the pathophysiology of many vision-threatening conditions such as age-related macular degeneration, polypoidal choroidal vasculopathy, central serous chorioretinopathy, and myopic chorioretinopathy [1]. Choroidal thickness is an important biomarker of choroidal health and reflects choroidal changes. The introduction of enhanced depth imaging spectral-domain optical coherence tomography (EDI-OCT) has enabled in vivo cross-sectional imaging of the choroid and accurate measurement of choroidal thickness [2]. In recent years, subfoveal choroidal thickness (SFCT) has received attention in the clinical setting for decision-making regarding the diagnosis, management, and monitoring progression of various choroidal and retinal diseases [3]. 

To correctly interpret SFCT and know the physiological range, it is essential to have normative SFCT data among different populations and to understand its related demographic, ocular, and systemic factors [4]. Several studies have investigated the normal distribution and associated factors of SFCT using OCT. Accordingly, the average SFCT has been reported in a diverse range from 206.4 to 354.0 μm [1, 2, 5–19]. However, most previous studies were hospital-based with small sample sizes; so, their results could not be generalized to the overall population. Moreover, most published reports were related to Western and East Asian countries, and there is a lack of information on the Middle East; this is while ethnicity and racial factors can influence retinal and choroidal thicknesses [16]. Most of the previous studies also had a wide age range and included a small proportion of the elderly. Due to the age-related changes in SFCT and the increased prevalence of retinal and choroidal diseases at older ages, it is useful to have specific population-based normative data for SFCT in this age group.

Some factors including age [1, 3, 15, 17–19], sex [6, 16–18], axial length (AL) [6, 16–19], and refractive error [8, 13] were found to affect SFCT in different studies. However, our knowledge in this field is limited because previous studies included only limited factors and many potential associations were overlooked. To obtain a more accurate picture of SFCT relationships, it is necessary to try to control the effect of confounders as much as possible by considering various potential factors and comprehensive multivariable analysis. According to the above, the present population-based report aimed to investigate the distribution of SFCT and its relationship with a set of demographic, ocular, and systemic variables in an Iranian elderly population.

## Methods

### Study design and sampling

This report is part of the Tehran Geriatric Eye Study (TGES); a population-based cross-sectional study that was conducted from Jan 2019 to Jan 2020 on the urban elderly population of Tehran, Iran aged 60 years and above. A multistage stratified random cluster method was carried out for sampling. The 22 municipality districts of Tehran were defined as strata. Next, the block map of each district was prepared and each block was labeled as a cluster. A total of 160 clusters were randomly selected proportional to size from all 22 districts and each cluster contained 20 individuals. After identifying clusters, a sampling team was sent to the related addresses and located on the southwest side of the selected block. The first household was considered as the head of the cluster. Then, by counterclockwise movement while selecting the next households, all individuals 60 years of age and above were invited to participate in the study after explaining the study objectives and ensuring the confidentiality of the information. The study participants were transferred to the examination site (Noor Eye Hospital) on a pre-determined day free of charge.

Once the study participants arrived at the examination site, an initial interview was arranged to collect demographic and case history information (history of ocular and systemic diseases, previous myocardial infarction (MI) or cerebrovascular accident (CVA), previous ocular surgery, use of ocular and systemic medications, and health-related behaviors including smoking and alcohol consumption). In the next step, the participant’s height and weight were measured by a trained person and the body mass index (BMI) was calculated using the following formula: weight(kg)/height(m)^2^. Then, experienced nurses measured blood pressure using sphygmomanometry (OMRON, HEM-2228-E, Kyoto, Japan) and took blood samples for laboratory tests.

### Ocular examination and OCT imaging

Preliminary optometric examinations included measurement of uncorrected distance visual acuity (UCVA) using a LED visual acuity chart (Smart LC 13, Medizs Inc., Korea) at 6 m (m), objective refraction using an auto-refractometer (ARK-510 A, Nidek Co. 42 LTD, Aichi, Japan), subjective refraction and recording the best-corrected distance visual acuity (BCVA).

Study subjects then underwent anterior segment imaging using the Pentacam HR (Oculus, Wetzlar, Germany) with a 50- image scan automated mode. Only measurements were considered valid that displayed ‘OK’ in the scan quality specification (QS) box. Information on central mean keratometry (mean K), central corneal thickness (CCT), anterior chamber depth (ACD), anterior chamber angle (ACA), and white-to-white distance (WTW) were extracted from Pentacam’s maps and recorded. Three high-quality AL measurements with a signal-to-noise ratio (SNR) above 2.0 were carried out using IOL Master 500 (Carl Zeiss Meditec AG, Jena, Germany) and the average of three measurements was recorded as the final AL.

Choroidal imaging was performed using SD-OCT (Spectralis, Wavelength: 870 nm; Heidelberg Engineering Co, Heidelberg, Germany) with EDI mode after pupil dilation using two drops of tropicamide 1%. To consider diurnal variation and circadian rhythm effect, all OCT imaging were carried out between 10 a.m. and 4 p.m., at least 2 h after waking up. Participant’s keratometry and refraction data were entered into the instrument software to estimate optical magnification. The OCT device was positioned close enough to the eye to produce an inverted image of the fundus. Automatic real-time averaging and eye-tracking features were used. Seven sections, each comprising 100 averaged scans, were obtained within a 5 × 30-degree rectangle centered on the fovea. The horizontal section passing through the center of the fovea was selected for analysis. The SFCT was defined as the vertical distance from the hyperreflective line of the Bruch’s membrane to the hyperreflective line of the inner scleral surface. The images were taken by a single experienced technician.

Finally, all study participants underwent anterior and posterior segment ocular examination by an ophthalmologist using slit-lamp biomicroscope (Haag-Streit AG, Bern, Switzerland) and a + 90 diopter (D) lens. The intraocular pressure (IOP) was measured using Goldmann applanation tonometry (GAT).

### Definitions

Diabetes mellitus (DM) was defined based on the participant’s self-report, antidiabetic treatment, or HbA1c level equal to or above 6.5%[20]. Systemic hypertension (HTN) was defined based on the participant’s self-report, systolic blood pressure ≥ 140 mmHg and/or diastolic blood pressure ≥ 90 mmHg, or use of anti-hypertensive medication [21]. Current smoking was defined as smoking at least one cigarette per day lasting for at least 6 months.

### Exclusion criteria

Exclusion criteria were myopia or hyperopia greater than 6.00 D, any retinal or choroidal disease detected in fundus examination or OCT images, history of retinal surgery or laser treatment, severe cataract (grade 3 based on WHO classification system), and poor-quality OCT image (quality index value less than 25 dB).

### Statistical analysis

The mean and 95% confidence interval (CI) of the SFCT were reported in the whole sample and by age and sex groups. The effect of cluster sampling was considered in calculating the CIs. Both eyes of participants were included in the analysis. One-way analysis of variance (ANOVA) and independent samples t-test were used to compare the mean SFCT across different groups of demographic (age, sex), ocular, and systemic variables.

To account for the inter-eye correlation, simple and backward-stepwise multiple generalized estimating equation (GEE) models were used to investigate the relationship between SFCT and different study variables. A *P* value < 0.05 was considered statistically significant.

### Ethical issues

#### Informed consent

was obtained from all participants. The principles of the Helsinki Declaration were followed in all stages of this study. The protocol of the study was approved by the Ethics Committee of the National Institute for Medical Research Development (NIMAD) under the auspices of the Iranian Ministry of Health (Ethics code: IR.NIMAD.REC.1397.292).

## Results

3310 0f the 3791 invitees participated in the TGES. OCT data was available for 1307 individuals. After applying the exclusion criteria, the final analysis was performed on 1060 eyes from 566 participants. Of these, 335 (59.2%) were female, and the mean age was 67.1 ± 6.14 years (range: 60 to 94). The mean SE was 0.34 ± 1.31 D (range: -5.38 to + 4.75).

**Fig. 1:**
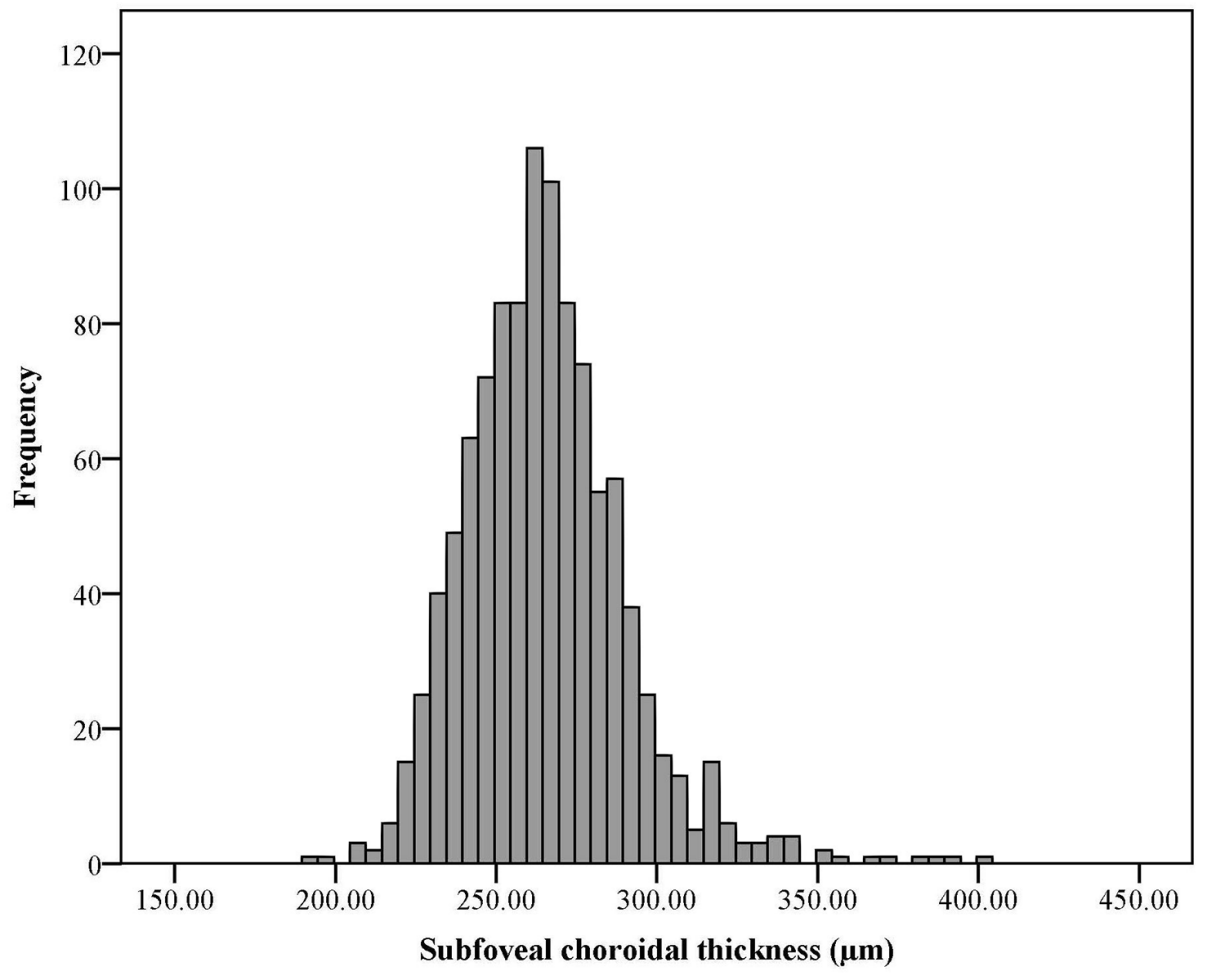
The distribution of subfoveal choroidal thickness in the elderly population

The average SFCT was 265.3 ± 25.9 μm (95% CI: 262.8-267.7) in the whole sample. Figure 1 shows the distribution of SFCT in this study. According to the results, the Skewness and Kurtosis of SFCT were 0.972 and 2.882, respectively. The 50%, 95%, and 99% percentiles of SFCT were 264, 308, and 344 μm, respectively.

**Fig. 2:**
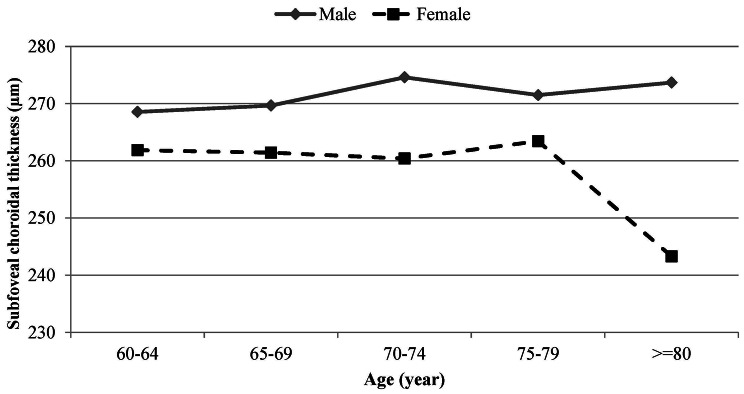
The pattern of age-related changes in the mean subfoveal choroidal thickness by sex

Table 1 shows the average SFCT by age and sex. The mean SFCT was 270.5 ± 23.7 μm (95% CI: 267.1–274.0) and 260.2 ± 26.7 μm (95% CI: 256.8-263.6) in males and females, respectively (*P* < 0.001). As seen in Table 1, SFCT changes with age were not linear, however there was a significant interaction between age and sex in the average SCFT. Figure 2 illustrates the pattern of age-related changes in the mean SFCT by sex. In males, the average SFCT increased with advancing age from 268.5 μm in the age group 60–64 years to 273.7 μm in the age group ≥ 80 years, while the average SFCT decreased with age in females from 261.9 μm in the age group 60–64 years to 243.3 μm in the age group ≥ 80 years.

**Table 1:** The mean, standard deviation, and 95% confidence interval of subfoveal choroidal thickness (µm) in elderly population by age and sex

	Total	Male	Female
	mean ± SD (95% CI)	mean ± SD (95% CI)	mean ± SD (95% CI)
60–64	265.0 ± 22.4 (262.1-267.8)	268.5 ± 18.7 (263.3-273.8)	261.9 ± 24.3 (259.0-264.8)
65–69	265.6 ± 26.7 (261.9-269.3)	269.7 ± 24.4 (264-275.3)	261.4 ± 27.9 (256.7-266.2)
70–74	267.7 ± 29.7 (261.8-273.7)	274.6 ± 29.6 (266.4-282.8)	260.4 ± 27.7 (254.1-266.7)
75–79	267.8 ± 24.6 (259.5-276.1)	271.5 ± 23.6 (263.3-279.6)	263.4 ± 24.0 (246.8 280.1)
>=80	258.0 ± 26.5 (242.6-273.4)	273.7 ± 31.0 (260.0-287.4)	243.3 ± 17.2 (222.9 263.7)
All	265.3 ± 25.9 (262.8-267.7)	270.5 ± 23.7 (267.1–274.0)	260.2 ± 26.7 (256.8 263.6)

*P*-value < 0.001 for comparison of mean subfoveal choroidal thickness between males and females as calculated by independent samples t-test

*P* = 0.513 for comparison of mean subfoveal choroidal thickness between age groups as calculated by one-way analysis of variance

**Fig. 3:**
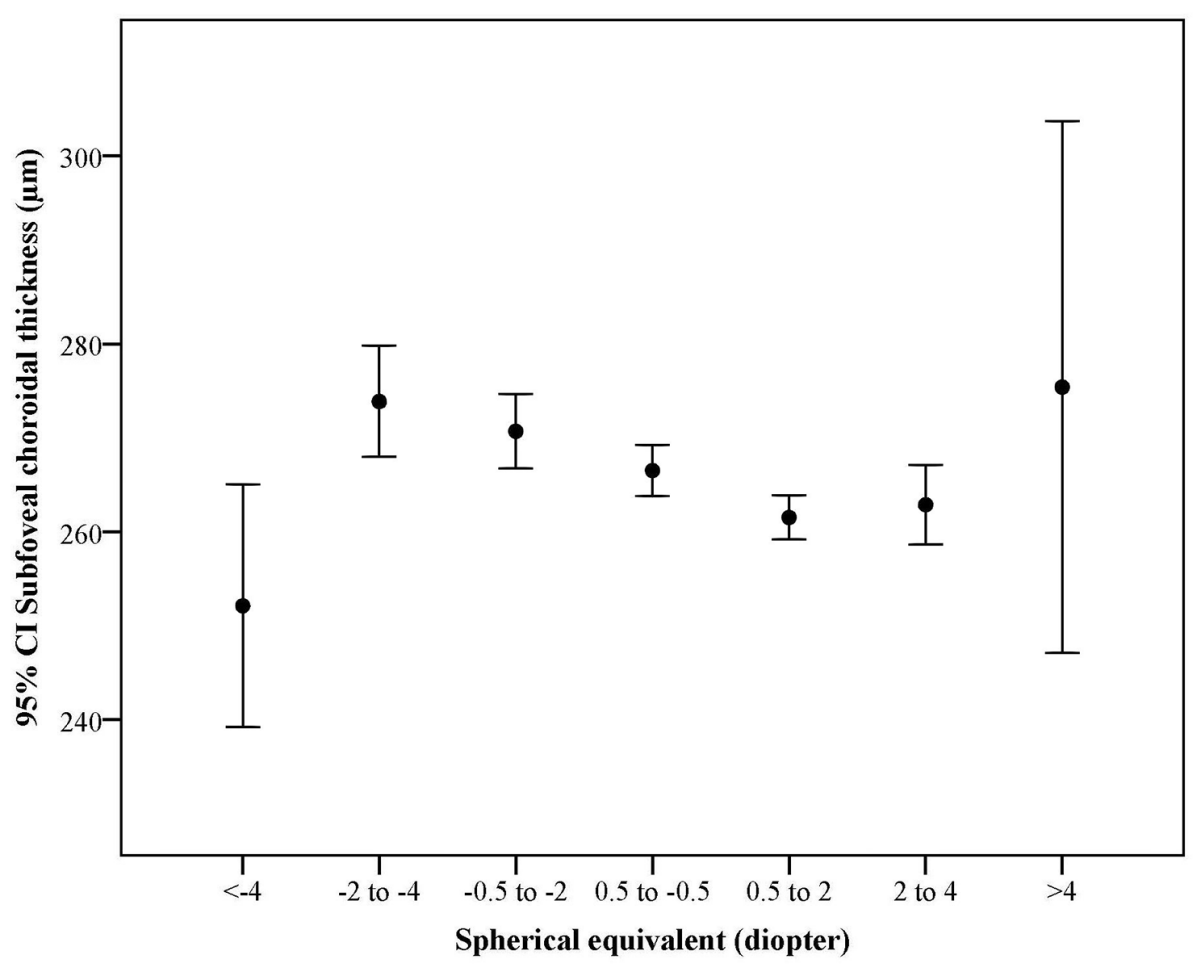
The distribution of subfoveal choroidal thickness by spherical equivalent

Table 2 shows the average SFCT based on DM, HTN, history of CVA, history of MI, current smoking, alcohol consumption, and crystalline lens status. Figure 3 illustrates the average SFCT by the severity of refractive errors. As seen in Fig. 3, the lowest average SFCT was observed in individuals with myopia equal or greater than 4 diopters.

**Table 2:** The mean, standard deviation and 95%CI of Subfoveal choroidal thickness (µm) in elderly population by general variables

Variables		Mean ± SD(95%CI)	*p*-value*
Diabetes	No	264.7 ± 24.1(261.9-267.4)	0.478
	Yes	266.8 ± 29.9(262.1-271.4)	
Hypertension	No	266.3 ± 22.4(260.7-271.9)	0.794
	Yes	265 ± 26.7(262.3-267.8)	
Cerebrovascular accident	No	266 ± 26.1(263.1-268.8)	0.017
	Yes	261.6 ± 24.7(256.6-266.6)	
Myocardial infarction	No	265.6 ± 26.0(262.8-268.3)	0.157
	Yes	263.3 ± 25.2(258.0-268.6)	
Smoking	No	264.9 ± 26.3(262.1-267.7)	0.622
	Yes	267.5 ± 23.4(262.6-272.4)	
Alcohol consumption	No	265.1 ± 25.6(262.6-267.7)	0.465
	Yes	266.6 ± 29(258.2-274.9)	
Lens status	Phakic	264.9 ± 23.5(262.7-267.2)	0.018
	Psodophakic	266.0 ± 30.7(259.0-272.9)	

*The *p*-value was calculated by linear regression

The relationship between SFCT with different study variables was examined using simple and multiple GEE models; the results of these models are shown in Table 3. According to the multiple GEE model, pseudophakia had a statistically significant direct relationship with SFCT [coefficient = 5.69 (95% CI: 1.55 to 9.83)], and history of CVA was significantly inversely related to SFCT [coefficient=-4.77 (95% CI: -9.82 to 0.28]. Moreover, there was a significant interaction between age and sex in the average SFCT so that with increasing age, the SFCT increased in men and decreased in women.

**Table 3:** Association of subfoveal choroidal thickness with study variables using simple and multiple generalized estimating equation (GEE) models

Independent variables	Simple model	*P*-value	Multiple model	*P*-value
	Coefficient (95% CI)		Coefficient (95% CI)	
Age	0.23 (-0.08 to 0.55)	0.148		NR
Sex (reference: male)	-9.75 (-13.58 to -5.92)	< 0.001		NR
Diabetes mellitus	1.52 (-2.68 to 5.72)	0.478		NR
Systemic hypertension	0.64 (-4.18 to 5.47)	0.794		NR
Body mass index	-0.36 (-0.78 to 0.07)	0.103		NR
Cerebrovascular accident	-6.26 (-11.41 to -1.1)	0.017	-4.77 (-9.82 to 0.28)	0.064
Myocardial infarction	-4.06 (-9.68 to 1.56)	0.157		NR
Current smoking	1.41 (-4.21 to 7.04)	0.622		NR
Alcohol consumption	2.55 (-4.28 to 9.37)	0.465		NR
Axial length	1.56 (-0.24 to 3.37)	0.089		NR
Anterior chamber depth	6.91 (1.06 to 12.75)	0.021		NR
Anterior chamber angle	0.15 (-0.04 to 0.33)	0.120		NR
Central corneal thickness	-0.01 (-0.07 to 0.05)	0.760		NR
Mean keratometry	0.69 (-0.43 to 1.82)	0.227		NR
White-to-white distance	1.81 (-2.36 to 5.98)	0.396		NR
Intraocular pressure	0.08 (-0.77 to 0.94)	0.849		NR
Pseudophakia (reference: phakia)	5.05 (0.85 to 9.26)	0.018	5.69 (1.55 to 9.83)	0.007
Age-sex interaction	-0.14 (-0.2 to -0.09)	< 0.001	-0.14 (-0.2 to -0.08)	< 0.001

CI: Confidence interval; NR: not retained (*P*-value > 0.05)

## Discussion

The present study examined the distribution and related factors of SFCT in a large sample of the Iranian general elderly population. The average SFCT was 265.3 μm in the studied population. Table 4 presents a list of previous studies that investigated SFCT using OCT. As seen in Table 4, the reported mean SFCT varies considerably from 206.4 μm in the study by Arnould et al., [19] in France to 354.0 μm in the study by Ikuno et al., [1] in Japan. The average SFCT in the present study was higher than the values found in China [15, 17], Malaysia [18], Singapore [16], and France [19] and lower compared to other countries.

**Table 4:** The reported average subfoveal choroidal thickness (µm) in different studies

First author	Country	Study design	Sample size (Number of eyes)	Mean age (years)	OCT device	Average SFCT
Ikuno et al. (2010) [1]	Japan	Clinical-based	86	39.4	Prototype HP-OCT	354 ± 111
Sanchez-Cano et al. (2014) [5]	Spain	Clinical-based	95	23.8	Spectralis SD-OCT	345.67 ± 81.80
Li et al. (2011) [6]	Denmark	University-based	93	24.9	Spectralis SD-OCT	342 ± 118
Rahman et al. (2011) [2]	England	Clinical-based	50	38.0	Spectralis SD-OCT	332 ± 90
Akhtar et al. (2018) [7]	India	Clinical-based	230	44.0	DRI-OCT-1	307 ± 79
Kim et al. (2014) [8]	South Korea	Clinical-based	286	40.18	Spectralis SD-OCT	307.26 ± 95.18
Moussa et al. (2016) [9]	Egypt	Clinical-based	129	36.85	DRI-OCT-1	300.87 ± 72.256
Margolis and Spaide (2009) [10]	USA	Clinical-based	54	50.4	Spectralis SD-OCT	287 ± 76
Erkul et al. (2013) [11]	Turkey	Clinical-based	123	47.47	Spectralis SD-OCT	280.23 ± 81.15
Manjunath et al. (2010) [12]	USA	Clinical-based	34	51.1	Cirrus HD-OCT	272 ± 81
Fujiwara et al. (2012) [13]	Japan	Clinical-based	145	45.7	Spectralis SD-OCT	265.5 ± 82.4
Pongsachareonnont et al. (2019) [14]	Thailand	Clinical-based	144	41.0	DRI-OCT-1	265.5 ± 74.2
Present study	Iran	Population-based	1060	67.1	Spectralis SD-OCT	265.3 ± 25.9
Ding et al. (2011) [15]	China	Clinical-based	420	49.73	Spectralis SD-OCT	261.93 ± 88.42
Song et al. (2020) [16]	Singapore	Population-based	2794	60.9	Spectralis SD-OCT	255.2 ± 102.6
Wei et al. (2013) [17]	China	Population-based	3233	64.3	Spectralis SD-OCT	253.8 ± 107.4
Gupta et al. (2015) [18]	Malaysia	Population-based	540	62.70	Spectralis SD-OCT	242.28 ± 97.58
Arnould et al. (2019) [19]	France	Population-based	1494	81.9	Spectralis SD-OCT	206.4 ± 83.0

Several factors may be involved in these discrepancies, including differences in age distribution, sample size, study design, type of OCT used, exclusion criteria as well as racial differences. As shown in Table 4, studies with a younger mean age reported higher average SFCT compared to those with an older mean age. There are increasing data from recent studies that indicate SFCT decrease with advancing age and this has been attributed to a decrease in vascular density, overall luminal area, and diameter of the choriocapillaris with age [22]. Hospital-based studies with small sample sizes also reported significantly higher average SFCT compared to population-based studies. This finding can be explained based on non-random sampling and the possibility of selection bias in the hospital setting; however, it should be noted that population-based studies had an older mean age compared to hospital-based studies. In a general view, the average SFCT in European countries [2, 5, 6], USA [10, 12], Egypt [9], India [7], and Turkey [11] was higher than reported in East Asian countries [14–18]. This finding could show the influence of ethnicity on the SFCT. However, the thinner SFCT in East Asian countries may also be due to the high prevalence of high myopia, a known risk factor for choroidal thinning [23]. Differences in exclusion criteria can also play a role in these discrepancies. For example, some previous studies excluded participants with a history of cataract surgery. However, in the current study, the history of cataract surgery was not considered an exclusion criterion due to the age range of the participants, rather it entered the model as an independent variable.

Although men had significantly higher SFCT compared to women and a significant relationship between sex and SFCT was found in univariate analysis, this association did not maintain in the multivariable model. Many previous studies including population-based studies [16–18] reported a significant relationship between thicker SFCT and male sex. Therefore, it seems that the relationship between sex and SFCT in previous studies may be due to the lack of effective control over confounders. There were no linear changes in the mean SFCT with age in the present study; changes were not noticeable from 60 to 79 years, but a significant decrease (about 9 μm) was observed after 79 years. Overall, the relationship between age and SFCT was not significant in either univariate or multivariable models, and this was contrary to previous studies that reported significant age-related choroidal thinning [1, 3, 13, 15, 17, 18]. When age-related changes in SFCT were analyzed by sex, an interesting finding emerged. In both sex groups, the changes were slight without a specific pattern (ascending or descending in different intervals) from 60 to 79 years. After 79 years, a significant decrease (20 μm) and a slight increase (2 μm) in the average SFCT were observed in females and males, respectively. These findings indicate that age-related changes in SFCT are sex-specific and choroidal thinning occurs prominently in females after the age of 79 years. This finding could be explained by the increased basal sympathetic tone and loss of parasympathetic tone in women at older ages [22]. The tone of the sympathetic and parasympathetic systems may have a significant role in maintaining the choroidal blood flow and thickness [22]. Experimental models have shown that stimulation of the cervical sympathetic chain results in frequency-dependent vasoconstriction and reduction in choroidal blood flow [24]. Similarly, in a pigeon model, stimulation of the parasympathetic Edinger-Westphal nucleus significantly increased choroidal blood flow [25]. The reduced choroidal thickness in aging women may also result from a reduced estrogen-dependent vasodilatory effect in the ophthalmic artery secondary to menopausal estrogen deficiency [26]. The slight increase in the mean SFCT in men after 79 years of age might be caused by the effects of some medications. For example, phosphodiesterase 5 inhibitors prescribed to treat erectile dysfunction in older men have been found to increase choroidal thickness [27]. Overall, this finding is important regardless of its exact etiology as it can partly explain sex differences in the occurrence of macular and choroidal diseases.

Pseudophakia was significantly associated with an increased SFCT, and this is in agreement with the results of previous studies that reported an increase in SFCT after cataract surgery in the short and long term [28–30]. The exact mechanism whereby cataract surgery affects SFCT is unclear and various theories have been proposed in this regard. Choroidal thickening after cataract surgery may be due to an inflammatory response. Surgical trauma leads to the release of prostaglandins in the aqueous humor and the disruption of the blood-aqueous barrier. Following impairment of the blood-aqueous barrier, other inflammatory mediators such as endotoxin, cytokines, and immune complexes accumulate in the aqueous humor; these inflammatory mediators pass through the vitreous to the retina, where they are responsible for a rupture of the inner and outer blood–retinal barrier [28]. Another mechanism for surgically induced inflammation is the increased expression of genes related to cytokine IL-1b, chemokines such as CCL-2 and SDF-1 and growth factors FGF and VEGF, which can disturb the blood-retinal barrier, retinal pigment epithelium, and choroid [28]. The third mechanism implies increased ocular perfusion pressure due to a decrease in IOP after surgery [30]. Another possible mechanism is increasing the amount of light entering the eyes, leading to metabolic activation and angiogenesis [29]. Finally, the measurement error during pre-operative evaluation should not be ignored. The effect of cataract surgery on SFCT could be of interest from the viewpoint of the potential connection between cataract surgery and late age-related macular degeneration; a debatable subject in ophthalmic research.

There is limited information regarding systemic determinants of SFCT in the literature. An interesting finding of the present study was the significant relationship between reduced SFCT and a history of CVA. About 75% of ischemic cerebral strokes originate from the internal carotid artery [31]. The vessels suppling the choroid (anterior and posterior ciliary arteries) are branches of the internal carotid artery; therefore, an ischemic stroke could affect choroidal circulation. First, vasoconstriction may occur in the choroidal vessels in response to decreased blood flow [32]. Subsequently, chronic choroidal ischemia may lead to the loss of choriocapillaries, choroidal atrophy, and a decrease in SFCT [32]. In support of this theory, Li et al. reported a significantly lower mean SFCT in patients with severe carotid stenosis compared to normal control subjects (230.32 vs. 251.86 μm) [33]. Vasoconstriction in choroidal vessels, and choroidal ischemia are involved in the pathophysiology and progression of many choroidal and retinal diseases [32]. Therefore, CVA could be hypothesized to be a risk factor for choroidal and retinal diseases, and further studies are recommended to investigate this issue.

The AL, DM, and HTN were not significantly related to SFCT in the present study, while some previous studies reported thinner SFCT in eyes with longer AL [6, 16–18] and in patients with DM and HTN [34, 35]. We believe that these controversies are possibly due to the differences in the exclusion criteria and metabolic characteristics of the studied populations. Since the present report aimed to determine the normal distribution of SFCT, subjects with high myopia were excluded because of possible chorioretinal atrophic changes. Although the average SFCT was lower in higher degrees of myopia (Fig. 2), applying this exclusion criterion may have led to an insignificant relationship between AL and SFCT. The lack of significant influence of DM and HTN on SFCT in the present study may result from better control or shorter duration of diseases. Longer duration of DM is an important risk factor for choroidal thinning [36]. Considering the age range of the participants, it can be expected that many had type 2 DM of short duration, while previous studies on younger patients probably included patients with type 1 DM of longer duration.

The present study has some limitations. Due to the cross-sectional nature, it is not possible to elucidate cause-effect relationships. Moreover, the lack of the severity of systemic diseases that can affect the SFCT (DM, HT) and not having a standardized definition of the use of systemic medication, caffeine, and nicotine are important limitations of the study.

## Conclusion

The normal values of SFCT in the present study can be used as a reference database for clinical and research purposes. Age-sex interaction, pseudophakia, and history of CVA were significantly associated with SFCT in the elderly population. It is recommended that these factors be taken into account when interpreting SFCT data.

## Data Availability

The datasets used and/or analyzed during the current study available from the corresponding author on reasonable request.
